# Colourism and law in the UK: a story of colonial indifference?

**DOI:** 10.3389/fsoc.2025.1510814

**Published:** 2025-06-04

**Authors:** Zainab Batul Naqvi

**Affiliations:** Manchester Law School, Manchester Metropolitan University, Manchester, United Kingdom

**Keywords:** colourism, coloniality, antidiscrimination law, hate crimes law, equality act 2010, skin tone

## Abstract

In this paper I explore legal and judicial responses toward skin tone and colourism in the UK. I argue that despite incidents involving colourism coming before the courts, there is no mention of this oppression in the legal framework. Colourism is invisible to the law and courts. “Colour” is included under the protected characteristic of “race” in the equalities and hate crime frameworks, but this is inadequate. This approach, I argue, reinforces the wider issue that the law is incapable of effectively addressing racism so measures to tackle colourism are even further beyond its scope. The law’s weaknesses here are emblematic of its indifference toward racially minoritised communities and their experiences. This colonial-inspired strategy of indifference is dismissive, dehumanising, and disdainful toward the vulnerable people and groups who are most affected by colourism.

## Introduction

In recent years, several TV personalities have faced public backlash around presenting documentaries about Black women’s experiences. The announcement that Leigh-Anne Pinnock of Little Mix would be fronting a 2021 BBC documentary on colourism and race in the music industry prompted severe criticism.^1^ Similarly, actress and singer Rochelle Humes received death threats after false rumours spread that she had replaced the presenter Candice Brathwaite in a Channel 4 programme about Black women and childbirth (O’Connor, 2021). The reason for this? Leigh-Anne and Rochelle have light skin and the privilege this affords them led to anger that they were perhaps chosen to present these programmes over Black women with darker skin because of their skin tone.^2^ Both incidents raised concerns about colourism, a prejudice in which racially-minoritised people with darker skin tones and phenotypical features associated more with people of colour face mistreatment and disadvantages both outside and within their communities (Phoenix and Craddock, 2022a, 2022b, 2024a). In this paper I argue that UK legal responses to colourism are non-existent and incapable of addressing this form of oppression. This oversight stems from the colonial indifference which surrounds the law perpetuating the dehumanisation that underpins general attitudes toward racially minoritised communities and racism in the UK.^3^ The law is incapable of dealing with racism in the first place, so it is little wonder that despite references to colour, colourism is invisible. This is a colonial hangover echoing imperial behaviour toward colonised people that limits the possibility of legal repair.

Colourism is well-documented all around the world, but outside of the Americas the scholarship remains underdeveloped (Dixon and Telles, 2017). The reactions to Leigh-Anne and Rochelle demonstrate that colourism is a social concern in the UK. Over 8 million people of colour live in the UK and yet, there has been very little attention to colourism. We have research undertaken by Phoenix and Craddock looking at the experiences of Black men (2022a); colourism in minoritised ethnic families (2024a); the complex interactions of skin shade with desirability (2024b) and an ongoing project on colourism among young people (2022b). They have further collaborated with others to develop a colourism scale (Craddock et al., 2022) and investigate the effects of colourism on body image and wellbeing (Craddock et al., 2023). In other work, Spratt (2024) has investigated light-skin privilege and poor health outcomes among Black British women. Whilst there is research on colourism and law in other jurisdictions like the US (see for example Jones, 2000), there is no legal or policy literature that explicitly looks at colourism in the UK. This includes mainstream law textbooks (e.g., Fredman, 2023), government commissioned reports focussing on racialised communities (Lammy Review, 2017) and independent reports around antidiscrimination law (Bi, 2022).

I start this paper with three novel conceptual arguments. First, colourism has been overlooked in law, so understandings remain underdeveloped in the UK. Second, this manifests the wider colonial indifference around the law’s failure to cope with oppressions against racially minoritised communities, including racism. Third, colonial indifference flattens out the experiences of racialised communities in the UK. I then provide an overview of where we try but fail to find spaces in law to address colourism. I follow this with a discussion of my methodology for my original and rigorous review of British judicial responses to colourism which involved examining over 200 cases.^4^ Finally, I share three thematic analytic arguments from my findings. First, skin tone appears throughout criminal justice processes to disadvantage racially-minoritised people. Second, there are loaded interactions around skin tone subsumed under race discrimination which fail to recognise the nuances of colourism and racism. Third, skin tone is used in particularly racialising areas of law like immigration as evidence for people’s identity. In all three situations, skin tone is weaponised by legal authorities to orientalise racially-minoritised people. We need an in-depth understanding of how legal institutions can use skin tone to oppress communities of colour. I am not calling for an immediate reform of the law. We need legal change, but this alone is insufficient. Instead, I expose what current legal responses tell us about the operation of legal power in relation to colourism and racism. The lack of acknowledgement of colourism is a black box: a box which if opened, will tell us about the complex inner workings of legal discourses and how they are maintained (Latour, 1987).

First, a note about my position in this project. I am a woman of colour with light skin who grew up in a South Asian community in the UK. I have witnessed incidents where those closest to me were subjected to colourist remarks. By the same token, I have received praise for my light skin which is just as problematic. My situation is mired in privilege – something that I have not spoken against enough. This is not a confession to absolve me of my guilt but to encourage my resolve to tackle this. My aim with engaging in this work is to express support, solidarity and care toward my people who move through the world with the pain of colourism and the devastating lack of attention to that pain. I am also conscious of recent interventions into the drive for these declarations of privilege in research. Gani and Khan (2024) persuasively argue that positionality statements in research are rooted in enlightenment philosophy. They were introduced for the white researcher to relieve themselves of guilt toward their non-white research subjects. They have also become a marker of authority leading to a narcissistic performance of radicalism (Hamati-Ataya, 2018). Through my small contribution, I hope that I can practise humility and learn how to recognise and end my complicity in colourism and its pernicious effects.

## Conceptual framing: colourism and indifference

I now turn to colourism as a concept to make sense of the law. I argue that part of the reason colourism has been overlooked is due to a lack of knowledge and understanding. What exactly are we missing? Several analytical categories that feature in my discussion throughout this paper need explaining first. Skin colour is one device for assigning us to a race. Race or categorising people for example as Black, Brown or white can be understood as a “floating signifier” which takes on different meanings at different times (Hall, 2021). It is a social rather than biological construct (Malkani, 2024). Race is not supported by objective physical characteristics and is not genetically inherent or a biological fact (Mirza and Warwick, 2022; Andrews, 2018). Ethnicity is often used interchangeably with race, but it was historically a way to describe shared identity based on “culture, descent and territory” (Mirza and Warwick, 2022, p. 8). Considering it synonymous with race is problematic because it equates the immutability of skin colour with the changing cultural norms that ethnic identity is based upon (Adébísí, 2019). Under colonial rule, this is exactly what happened as colonisers used skin tone to differentiate between ethnic groups to create divisions within communities. Finally, whilst I do not engage in discussions of nationality, in the UK this includes the citizenship of an individual (Equality Act Explanatory Notes, 2010, para 50).

Colourism involves the mistreatment and oppression of racialised people with darker skin tones and phenotypical features associated more with people of colour compared with lighter-skinned members of their communities. The “colour” in colourism is shorthand for a complex entanglement of “perceived physiognomy, behaviour, and culturally-transmitted expectations and assumptions” (Harris, 2008, p. 60). While skin colour is one device for assigning us to a racial category, for colourism the social meaning afforded to skin tone itself leads to the disadvantageous treatment (Jones, 2000). Further, colourism occurs within racialised communities where members with darker skin are mistreated by members of the same community. And it can be inflicted by those from different racialised communities too. The terminology around skin is complex in this context so I adopt the clumsy distinction of using skin tone when referring to colourism-related discourses and skin colour when looking more broadly at race.

Some scholarship discusses colourism as motivated by a desire for proximity to whiteness (Bhattacharya, 2012). Through this, we can trace the development of colourism back to European colonialism and the idealised physical appearance of the coloniser (McClintock, 2004). Taking this approach, it is much easier to draw the connection between colourism and racism. “Colourism” stems from the physical manifestations of our “race” which leads to colourism essentially being a form of racism. However, this fails fully to account for the colourism that happens within communities of colour, and it sidelines the skin tone hierarchisations predating European imperialism (Cleland et al., 2007; Shevde, 2008; Tate, 2015). So, whilst we can argue that European colonisers might have exploited this skin tone hierarchy and encouraged it as a desire for whiteness (Anjari, 2023), we need to avoid reducing colourism to only wanting to be white and a sub-category of racism.

This oversimplification has been noted in other jurisdictions like the US. Jones (2000) tells us that despite colourism’s longstanding presence in the US, it is overshadowed by racism. The courts are unaware of it or minimise its importance. For example, in *Ali v. National Bank of Pakistan*, 508 F. Supp. 611 (S.D.N.Y. 1981), where a Pakistani bank employee brought a claim for discrimination against another Pakistani colleague based on skin tone, it was decided that imported biases brought over to the US from other cultures could not establish a new “protected class” (Nance, 2005). Colourism is not something the US legal imaginary ignores or fails to understand – it has outright rejected it as “imported.” The same imperious attitudes are not out of place in the UK. Like racism, there is little recognition of colourism as part of structures which are maintained by supposedly neutral laws and policies that disadvantage its victims. This means that colourism (when it is acknowledged) is only viewed as a result of individual behaviour that is prejudiced and discriminatory (Reece, 2019). Legal responses to colourism are more than just a benign ignorance – there are active processes framing it as imported or migrated into the US. These responses are not harmless. There could be a strategy behind this to designate colourism a foreign oppression which the courts and law need not concern themselves with. It is an example of orientalist “blackboxing,” where the courts state “facts” and authorise views about colourism and those that suffer with it as “other” (Said, 1978). These people and their oppressions are not ours; they are other and from elsewhere. A black box is used to encase a contested process or practice that has become part of common knowledge to maintain the fiction that it is a fact and cannot be challenged (Riles, 2005).

Such distancing denotes the legal strategy of “indifference” which arises in colonial administrations but can be seen in other contexts. My starting point is that indifference is found in the *absences* rather than the *presences* in law. It makes sense of the invisible, the silent, the missing. It is hidden in the black boxes throughout the law (Valverde, 2009). The law has often been depicted as technical, neutral, objective and devoid of any of the messy biases that humans have. This attitude has underpinned the use of law in colonial rule for centuries. The courts’ focus is supposedly on the technical application of those rules rather than the ideological issues that obscure law’s role in building empire and constructions of race into the present day (Muir Watt, 2017; Garland et al., 2022). The law has always been used as a powerful tool to enforce colonial order and impose norms onto colonised communities. Law has shaped the ways that racialised people are treated today but black boxes work to diminish our awareness of this.

How does indifference fit in here? Indifference suggests a lack of knowledge and concern; a sense of emptiness or nothingness (Tester, 2002). This might be innocuous. If we are indifferent, it is because we simply do not know enough to have an opinion. We might be indifferent to colourism because we have never encountered it. And yet, as I have argued elsewhere, indifference has dangerous effects (Naqvi, 2023). Indifference is not just being unaware but also being uninvested and unbothered. This attitude of not caring enough is what leads to minoritised groups becoming invisible (Passini, 2019). To not care can sometimes take as much effort as it does to care. It is not as empty as we think; underneath the surface it is brimming with feelings drowned out by the monochromatism of being indifferent (Tester, 2002). Indifference subjugates those sensations and requires an active investment by the person practising it – it is intentional, political and harmful (Granzow, 2020).

Indifference derives from sites of power like law and expresses a cultural and emotional distance resulting in impassive, fake concern from those in power. It shows up in everyday mundane administrative and governance processes. We do not notice indifference because it is lurking underneath every surface. At its core, indifference facilitates the dehumanising of those deemed unimportant. The most marginalised and minoritised of us. The quickest way to cast something as unworthy of concern in law is for it not to exist in the first place. The law is indifferent to colourism because it is not important to the people who matter; therefore, it does not exist. This legal strategy of indifference, of stripping away the common humanity of its victims flattens out the experiences of racialised people and communities in the UK and the same attitude is prevalent in legal responses to racism. The black box surrounding colourism acts as a form of jurisdictional control which reveals how the coloniality of law operates to see or unsee; to react or remain unmoved by communities associated with the colonised. As we saw with the case of *Ali* earlier: colourism is something that affects the “other,” the foreigner who is outside of the mainstream legal gaze. If you suffer with colourism, it is not important to the law, which means you are not important to the law. And if you are not important to the law, then what is the law saying about you? That your suffering is non-existent? That you are non-existent?

## Spaces in the law?

There is no legal attention toward colourism in the UK. This discussion of the law could easily end here but what about skin colour? Could laws covering skin colour provide space for mistreatment based on skin tone? We have to resort to looking at race and its association with skin colour so there are two possible legal areas for this: hate crimes and hate speech or antidiscrimination law. Starting with hate crimes and hate speech, in England and Wales these criminal offences are set out across four different statutes: the Public Order Act 1986; the Crime and Disorder Act 1998; the Football (Offences) Act 1991 and the Sentencing Code.^5^ Hate crimes are treated more seriously than offences which do not involve hostility toward a protected characteristic (race, religion, sexual orientation, disability, transgender identity). First, by prosecuting them under the discrete category of aggravated offences which have a higher maximum penalty (Crime and Disorder Act 1998, ss28-32) and second, by prosecuting them under existing criminal offences but subjecting them to enhanced sentencing to increase the maximum sentence (Sentencing Code, s66). The aggravated offences only apply to racial and religious hostility, but enhanced sentencing applies to all of the protected characteristics.

For hate speech, there are also specific offences of “stirring up” hatred in relation to race, religion and sexual orientation (Public Order Act 1986, Pts 3 and 3A). The “stirring up” offences only criminalise conduct (use of words, material or behaviour) targeting entire groups rather than specific individuals. For the purposes of hate crime laws, a racial group is “a group of persons defined by references to race, colour, nationality (including citizenship) or ethnic or national origins” (Public Order Act 1986, s17; Crime and Disorder Act 1998, s28(4); Sentencing Code, s66(6)(a)). “Colour” could cover colourism-based offences, but it is not defined. Combining this with my brief examination of over 200 racially-aggravated hate crime case reports where I was unable to see any instances of skin tone being addressed, the inclusion of colour in the hate crimes framework must be limited to describing a person’s socially-constructed race (e.g., Black, Brown etc).

This is supported by British equality or antidiscrimination laws. The Equality Act (2010) requires public bodies to have due regard to the need to eliminate unlawful discrimination, harassment and victimisation and promote equality of opportunity for people that experience inequalities and discrimination on the basis of their age; disability; gender reassignment; marriage and civil partnership; race; religion or belief; sex and sexual orientation (Fredman, 2023). Again, there is no reference to colourism but the explanatory notes for the Act tell us that the protected characteristic of race under Equality Act (2010), s9 includes “colour, nationality and ethnic or national origins” (Equality Act Explanatory Notes 2010, para 47). Similarly, people who have or share characteristics of colour, nationality or ethnic or national origins can be seen as belonging to a particular racial group (Equality Act Explanatory Notes 2010, para 48). And most illuminating of all, we are told: “Colour includes being black or white” (Equality Act Explanatory Notes 2010, para 50).^6^ So, equality law even includes discrimination for being white which speaks volumes about the race power relations at work (Song, 2014).^7^ This binarisation reinforces the essentialism of race in the law: if you are not white, you are only Black so all racially-minoritised people are collapsed into the category of Black (Lugones, 2010). Arguably, if colour “includes” Black or white, there is room for a more expansive definition of colour but there has been no application of Equality Act (2010), s9 to a claim of discrimination, victimisation or harassment based on having a darker skin tone compared to a person with light skin. The claims focus on skin colour as the physical manifestation of race, for example, being Black rather than being a Black person with a darker skin tone who is being discriminated against compared to a Black person with light skin. This is where we see the indifference at play: by limiting understandings of skin colour, there is a lack of interest in truly grasping the experiences of those subjected to racism and colourism. Legal actors do not care and never have because it does not touch them.

More generally, there are limits to including new categories within the existing protected characteristics of the Equality Act (2010). For example, when claims have been brought for menopause-related discrimination or harassment as a form of sex discrimination, the Act has not helped (James, 2024). There is no indication that colourism was on the minds of the drafters either which means it would be difficult to fit it within the characteristic of race. In another more relevant example, Equality Act (2010), s9(5)(a) provided for “caste” to be added as “an aspect of” race in the future. This later became a duty in Enterprise and Regulatory Reform Act 2013, s97. Not long after, in *Tirkey v Chandhok & Anor Appeal No. UKEAT/0190/14/KN*, it was decided that even though “caste” as an autonomous concept did not come within Equality Act (2010), s9 under the protected characteristic of race, many of the facts considering caste might be capable of doing so since “ethnic origins” under the Act had a wide and flexible ambit. This provided the first instance of an existing protected characteristic being potentially expanded. Further, it provided for the possibility of an intra-racial claim. The claimant and her employer respondents had the same racial background, but the claim of mistreatment was rooted in caste-related oppression. This is important when thinking about the possibility of a colourism-based claim against members of the same racial group. However, casteism and colourism are not analogous. Further, Waughray and Dhanda (2016) argue that the decision in *Tirkey* was created by the wider climate brought about by the 2013 duty. There is no such legislative backing for colourism, so expanding the “colour” element of the race characteristic is less feasible.

And then there is the bigger issue of our expectations. Are race-related hate offences and discrimination the same thing as racism? We can find race-based mistreatment being explicitly denounced in official legal practice publications such as the Guide to Judicial Conduct (2023); Equal Treatment Bench Book (2024) and the Judicial Diversity and Inclusion Strategy (see Monteith et al., 2022). These guides set standards for judges to challenge racial bias and prejudices in the courtroom and beyond. And yet, this is all framed around racial bias and prejudice rather than racism. These discursive moves to never name racism are threaded throughout the law. Race discrimination and hate offences are not synonymous with racism, especially structural racism (Atrey, 2021). Structural racism refers to how racism is integral to social, cultural, political and economic processes and institutions (Bonilla-Silva, 1997, 2021). Racism is not only found in isolated incidents, it is foundational to law and society. There is no substantive test or link between legal understandings of race discrimination or race-based hate crimes and structural racism (Atrey, 2021). Race-based discrimination, or the less favourable treatment and particular disadvantaging of people because of their race, is prohibited by the Equality Act (2010).^8^ However, we are told multiple times in *R v JFS* (2009) UKSC 15 that being guilty of race discrimination should not be interpreted as being “racist” as that word is generally understood (para 9). So, there is no indication that race discrimination in legal terms can be interpreted as racism (Atrey, 2021). There seems to be an explicit desire to decouple race equality law from antiracism. If the law does not even want to fully tackle racism in its structural forms and has set limits on its ability to do so (Malkani, 2024), there is little hope it would deal with colourism in a meaningful manner. The Equality Act (2010) is a misdirection. It gives us the impression that implementing it will achieve equality (Ahmed, 2012). But shying away from acknowledging the racist underpinnings for race discrimination means it is a half-hearted measure.

The same can be said of the hate offences law framework. Does prohibiting racially-aggravated offences and hate speech actually target racism? Again, terms like “racial” or “race” form the backbone of the legislation rather than “racism” (Goodall, 2013). And whilst the term “racialist chanting” in the Football (Offences) Act 1991 is interpreted by the Crown Prosecution Service (2022) as “racism,” the discursive distancing in the text of the law remains. The law enacts its indifference against racism, especially the structural forms by using adjacent terminology like race, racial hostility, prejudice and bias to obscure its real condition of being constituted by racism itself (Fitzpatrick, 1987). This indifferent approach is tokenistic: the law might end up treating some of the symptoms of racism through antidiscrimination and hate offence frameworks but is never able to escape its complicity in the structural racism that runs deeper. Like people who suffer because of racism, those oppressed by colourism would be failed if we solely relied on existing legal responses and frameworks to counter it.

## Note about methodology

Before sharing my analytic arguments, I explain my methodological approach. I looked at legal judgments from around the UK. I initially searched for these judgments on the legal database Westlaw using the search term “colourism.” However, this yielded nothing. So, I used the more expansive “skin colour” since there is no mention of “tone” in the legislation. I focussed on court judgments because as active agents who produce and reproduce discourses that reflect the values of the society they live in, judges tell us a lot more about what the law is intended to achieve and how it is used (Naqvi, 2023). I undertook a thematic analysis of the reports to look for repeated patterns of meaning about the ways that skin tone is viewed in the courts (Braun and Clarke, 2006). It was very difficult to find any reference to colourism in the case law. Instead, I point to the gaps or absences which suggest the artificial emptiness of indifference guiding the courts around colourism. The select examples of the themes from my analysis show where colourism and skin-tone related oppression are present but are not recognised. The examples are also tangled up with racism so there are no standalone incidents of colourism. There is no way for colourism to come up as an autonomous issue, so we are unlikely to see examples of it that do not overlap with racism. This paper and my analysis are therefore limited by the equation of colourism with racism. Despite examining cases up until 2022, many of the key findings are from cases in the earlier 2000s so this does not tell us everything we should know about legal and judicial responses to colourism in the UK. There is a need for more legal research which centres those affected by colourism. My aim is to raise awareness of just how desperately we need collectively to unpick this lack of legal engagement.

## Colourism in the courts?

### Skin tone and criminal law and justice

My first theme concerns criminal law and justice processes. There is no mention of colourism but there are repeated references to skin tone made by criminal justice system actors. Skin tone is a means of identifying people whether suspects or victims. As a result, the criminal justice system creates the ideal conditions for colourism-based oppression revealing the operation of legal power against racially-minoritised people:

‘For example, the deceased himself was variously described as an Asian man, a man of light skin colour who may have been Turkish in origin, white skinned with short, light coloured hair, and indeed white.’ (*Regina v Gifford George Mullings, Andre Anthony Morgan* [2004] EWCA Crim 2824, 2004 WL 2935850, para 13)

**‘**The point taken was that the appellant has a distinctive skin colour, namely that of a mixed race black but light-skinned man, whereas all the police officers, including DC Curzon, were adamant that the man they saw on 8th August was a black-skinned West African/ Caribbean and not a mixed race light-skinned man.’ (*Regina v Tunde Abiodun* [2005] EWCA Crim 09, 2005 WL 62280, para 24)

These two excerpts illustrate how skin tone is produced during criminal investigation and prosecution processes. The first concerns the victim of a murder whilst the second refers to an appellant who is appealing their conviction. In both cases as well as many others, the “skin colour” of the parties involved or affected, features heavily in the evidence. It is taken for granted that these descriptions are helpful, but in both cases, skin tone is contested which means the identity of the “subject” is unclear. How helpful are these descriptions? And how much do they work against the person being described? This might provoke the question of how we should expect people to be described in an investigation. If I cannot include a description of someone’s skin tone in my eye-witness account, then surely my account is incomplete? I am not expecting that this will change, and it is beyond the scope of this paper to propose an alternative. However, I want to cast light on the potential implications of these uses of skin tone.

In the first instance, whilst colourism is neglected in law, skin tone is not invisible in the UK legal gaze (White, 2015). There are clearly situations it is considered useful to distinguish between people. That being said, if skin tone does appear in law as a descriptor, who benefits from this and who suffers? I argue that mentions of skin tone in this context result in greater disadvantage to racially-minoritised people. I look to other jurisdictions where this has been researched to demonstrate further. For example, in their study of the association between skin tone; Afrocentric facial features; and criminal punishment in two Minnesota counties, King and Johnson (2016) found that the darker skin tone and Afrocentric facial features of offenders were associated with harsher sanctions. There are further US and Canada based studies reaching similar conclusions: members of racialised communities with darker skin are worse off than their lighter-skinned counterparts in encounters with the police and law (White, 2015; Lam and Bryan, 2021). This displays the colourism at play in and around the criminal justice system in other countries but what about the UK? Unsurprisingly, there is not much to go on.

Looking at the starkest encounters around stop and search powers, Bowling and Phillips (2007) observe that there is evidence police officers use skin colour when deciding whether to stop and search, rooted in stereotypes about the involvement of Black people in crime. However, this is based on the use of skin colour to identify the socially-constructed race of “suspects” rather than skin tone. Others argue that the police are “policing race” which indicates racialised communities are targeted for policing based on race rather than the tone of their skin (e.g., Barrett et al., 2014). Within the system, the Crown Prosecution Service (CPS) has reviewed racial biases and established a “racist incidents” monitoring service (HM Crown Prosecution Service Inspectorate, 2002; John, 2003). The Lammy Review (2017) which shares the findings from a purportedly independent review investigating the treatment and outcomes for racially-minoritised individuals in the criminal justice system commended the CPS for its diverse workforce and openness to scrutiny. But these enquiries have still found evidence of racial discrimination and biases. We see again the prominence of “discrimination” and “bias” which raises the question of whether they captured the experiences of racism happening at a structural level. There remains no reference to colourism by the government or official bodies like the police and CPS.

Using skin tone to identify people disproportionately affects racially-minoritised people. Even when there are no specific tones used, it is still harmful. In the *Abiodun* case above, the defence argued that the appellant had a distinctive “skin colour” which was lighter than the description of the police officers. Despite this being mentioned in the facts of the appeal, it is not dealt with in the judge’s examination. This was important enough to feature in the case facts yet is then left hanging like a loose thread. The weaponisation of skin tone and the ways in which it is unreliable for identifying people is completely ignored. But it is vital. It changes lives, it can harm lives. This lack of concern for the role of skin tone is the epitome of indifference. During these criminal justice processes, skin tone is taken as material fact for the police and courts – it forms part of the persuasive evidence. This acceptance of skin tone descriptions exposes the indifference to colourism hidden under the surface of the law. When we open the black box around colourism and skin tone, we see how the law maintains the racialising system designed to harm the most minoritised communities in the UK. It becomes even more apparent in this further example from a Scottish case:

‘The undisputed facts found by the sheriff were that the appellant was brought to Partick Police Office in custody to be placed in an identification parade. The police had secured the attendance of nine men of various ages and descriptions as stand-ins. The appellant's solicitor expressed a dissatisfaction with the stand-ins on the ground that they were white and pale skinned, while the appellant was of mixed race and dark skinned.’ (*Paul Murray Wright v Her Majesty's Advocate* 2006 S.C.C.R. 455, para 4)

Here, Paul Murray Wright who had been charged with robbery and assault challenged the admissibility of evidence from his identification parade. The appellant’s solicitor raised objections at the time of the identification parade regarding the differences in appearance between the suspect and the nine stand-in participants. Amongst those objections was that the stand-ins were all white whilst the appellant was a mixed-race man with darker skin. He challenged the admissibility of his identification parade evidence, but it was pushed on to be considered at his full trial because the sheriff did not think that they had sufficient information to do justice to the issue. He appealed this arguing that the parade had been unfairly conducted and should not be put before a jury at full trial. Ultimately, the appeal was rejected because the sheriff was right to think that they could not do it justice. I was unable to find the full trial judgment but there is an online news article about a Paul Murray Wright convicted of assault and robbery at Dumbarton Sheriff Court in May 2007 (Currie, 2008). The appellant made it into the news for breaking free from prison for around 4 minutes. This article tells us that he was convicted and so his appeal against the evidence probably had minimal effect on the outcome.

The first identification parade in England and Wales reportedly took place in 1860. Davies and Griffiths (2008) argue that despite improvements, identification parades still lead to errors in identifying suspects. As far back as 1976, the Devlin Report recommended that these parades be investigative rather than evidential (Devlin, 1976). They are already mired in weaknesses and injustices. One of the biggest issues stems from the differences in appearance that can occur between a suspect and the stand-ins in the parade. Code D of the Police and Criminal Evidence Act 1984 states that “The identification parade shall consist of at least eight people (in addition to the suspect) who, so far as possible, resemble the suspect in age, height, general appearance and position in life” (Code D, 2023, Annex B(c)(9)). At least legally, the other people in the parade should be similar in appearance which presumably includes skin tone but how well this is ensured in practice is not clear.^9^ Imagine the effects of being in a parade with nine other men who have a different race and skin tone to yours. It would be difficult to find a clearer expression of both racism and colourism in the criminal justice system – neither of which are being admitted or prevented. Here, the disparities between the appellant and stand-ins were a marker of difference in race but there were separate implications and social meaning to the difference in skin tones. It was not just that the appellant was mixed race but that his skin tone was darker. We cannot overlook this added layer of difference.

But when he challenged the admissibility of this evidence, the courts repeatedly said that there was not enough information available to deal with it before trial. Here, we see the Sheriff and courts plead ignorance – we do not know enough; therefore, we must be indifferent – we accept the law as it is and are not willing to open the black box. This emptiness in the attitudes of the criminal justice system actors reveals so much. It shows us the same orientalist lack of concern reserved for colourism in the US. It is not our problem, it comes from outside of our community, it does not make a difference to the case against this defendant, and it does not exist to us. The repeated dismissal of the appellant’s challenge encompasses an implicit denial of the racist and colourist processes underpinning the identification parade. This emphasises the dehumanisation of people on the receiving end of the oppressions embedded into the criminal justice system. Their oppression does not exist and neither do they fully exist as whole humans along with their skin tone. Because if your skin tone is not visible to the courts and legal actors, then you cannot be visible to them either.

### Skin tone and antidiscrimination

Earlier, I discussed how skin colour is presented in the British equality law framework. I now look at how this operates in practice by exploring the way skin colour can come up in antidiscrimination cases in subtle yet loaded ways. In *Diem v Crystal Services plc* [2006] All ER (D) 84 (Feb), the claimant, Anita Ho, complained about the behaviour of the employment Tribunal Chairman during her original race discrimination claim hearing:

‘At the Employment Tribunal hearing, whilst I was giving my evidence, the Tribunal Chairman, Mr S M Duncan, questioned me at some length as to why I was claiming to be non-white. In this context, he said that my skin colour was 'as white as the English'. Whilst he made this statement, he looked at me and used his finger to point at the skin of his other hand to stress the point. In fact, my complaint was not based on any claim that I was 'non-white', but on the fact that I am Vietnamese.

The chairman's comment and behaviour took me by surprise. When I did not answer, the chairman said to me: 'Your skin looks whiter than mine.' I felt pressurised to admit that my skin was white.

I felt that the chairman was mocking my appearance and perhaps my make-up was too white so that I looked like a clown or a geisha girl…’ (*Diem v Crystal Services plc* [2006] All ER (D) 84 (Feb), paras 4-6)

The claim was based on her identity as a Vietnamese woman, but the Chairman’s comments bear deeper examination. Starting with questioning her at length about why she was “claiming to be non-white.” This is an attempt to dismiss the race discrimination that she had endured during her employment. She is “claiming” to be non-white which means her honesty about both the mistreatment and her identity are being doubted. The Chairman is discrediting her and the discrimination. It gets worse with the Chairman then using the reductive and offensive arguments that her skin is “as white as the English” and whiter than his. The Chairman deliberately rejects the social construction of race and skin colour to humiliate the claimant. He is shaming her into admitting that her skin is white to imply that her claim is frivolous.

The comment about her skin colour being as white as English people, ensures the ideal of being white English is pitted against the claimant to make her feel inferior. Her skin colour is weaponised by the Chairman to highlight that her skin might be white and therefore her claim is unimportant but this acts at the same time to reinforce just how different and “other” she is. The claimant is forced to admit that her skin is white and that she has therefore not suffered any discrimination. I argue that the Chairman’s statements and gestures are a form of racial gaslighting. Abu Laban and Bakan (2022) posit three dimensions of racial gaslighting. First, that it involves the denial of lived experience and reality for racially-minoritised groups and individuals. Second, that it operates to maintain the status quo and is directed at those with less power. And third, that it involves a form of blaming the victim, typically by pathologising or treating as abnormal those who resist their oppression. In this case, the Chairman denies the claimant’s lived experiences and reality of discrimination; she is at the receiving end of this denial; and she is the one that is interrogated and pressured into admitting her skin is white. She is held responsible for making an unserious claim of race discrimination and therefore treated as unreasonable. This is what indifference looks like in law. The legal actor does not care and makes her feel at fault.

The final statement of this passage is also revealing. The claimant felt that her appearance was mocked which made her feel conscious of her makeup being too white so that she looked like a clown or geisha girl. Both of these descriptors: clown and geisha girl are associated with negative stereotypes, one of which concerns the appearance of the white makeup that they apply. Both clowns and geisha are performance artists. They apply makeup as part of their performance. Performance and entertainment are significant in the context of this case. When the claimant is made to feel that she has worn white makeup that has affected perceptions of her and the claim she is making, she is made to feel like a performer. Someone playing the role of a race discrimination victim. The Chairman has pushed her into feeling like a performer and not a victim through the emphasis on her skin colour compared with his. This imposes a narrative on her. She loses control of her story and experiences in the face of his prolonged questioning on her skin colour which should have no bearing on the claim.

The claimant’s concern about being seen as a geisha girl expresses an implicit fear of being associated with the orientalist stereotype of a geisha. Geisha are educated women and artists. They have been well-respected for their skills and performances for centuries in Japanese culture (Skotadi, 2013). They have gained attention through the years as the embodiment of beauty ideals in Japan and their white skin contributes to this (Yoshikawa, 2021). However, due to encounters with the white world including the runaway success of Arthur Golden’s *Memoirs of a Geisha* which was a fictional account of a geisha’s experiences, they have been exoticised in ways that misinterpret their profession as a form of prostitution.^10^ This has left a lasting impression with geisha being forced to clarify to white customers from the West that they are not prostitutes (Yoshikawa, 2021). For this analogy to present here, we can see a fear of the consequences of the Chairman’s words. He has made the claimant fear that she seems like a performer associated with a group that is at risk of being ostracised because of misconceptions about its craft. A craft which reflects the ideals of a particular skin tone.

The Chairman of the Tribunal claimed that it never crossed his mind that the claimant looked like a clown or geisha girl. He also argued:

‘…the Claimant did not give the impression of a person who was humiliated, embarrassed or mocked and further that in no way were any of his questions or comments intended to be offensive, nor did he believe that the Claimant was in fact offended’ (*Diem v Crystal Services plc* [2006] All ER (D) 84 (Feb), para 12)

In his view, the claimant did not seem humiliated or offended by his questions. This weak response focusses only on his perceptions of the situation. It is a continuation of his attitude from the start: that his questions and comments are legitimate. Since the claimant did not seem offended, he is absolved of responsibility. This is another demonstration of indifference – he does not believe she was offended which means he is ignorant of it and therefore indifferent to it. The claimant’s feelings do not exist, because his gaze as a white man in a position of authority does not capture them.

The appeal was upheld but part of the decision states the following:

‘We find that the fair-minded observer would conclude that the remarks made were likely to cause the Claimant to feel unsettled, humiliated and embarrassed…We accept…that the position is not improved by the chairman’s comment that he did not believe that the Claimant was in fact offended; although equally we accept that it was not the chairman’s intention to cause offence’ (*Diem v Crystal Services plc* [2006] All ER (D) 84 (Feb), para 26)

Even whilst accepting that the chairman’s comments were likely to cause the claimant to feel humiliated, the appeal tribunal still stated that the chairman did not intend to cause offence. This is a back handed victory. On the one hand, the claimant’s feelings are recognised, but the chairman was not intentionally offensive. In this way, the chairman’s behaviour and the blame are diminished. There is no real challenge to his actions, he did not intend to cause harm and so there are no real consequences beyond upholding the appeal. It is treated as an isolated incident, an exception that does not happen every day which denies the structural conditions that have led to this situation. The weaponisation of skin colour against a racially-minoritised person does not happen in isolation – but it appears this way because it is obscured by the black box that contains this process (Valverde, 2009). The indifference to the ways that racially-minoritised people are treated permeates the courts and legal responses to skin colour. This may not be a clear example of colourism in the courts, but it does showcase the lack of sensitivity, nuance and awareness of the ways that skin colour and tone present in the courts. It further illustrates how poorly the law and legal actors deal with race discrimination. And if this is happening in race discrimination legal processes, we can imagine how structural racism would be approached. Overall, it represents the legal strategy of indifference being deployed against racism and colourism. It is deliberate and invested: a form of colonial control.

### Skin tone and evidence of ethnicity

The final presentation of skin tone in the courts I share is visible in asylum case law where it forms part of the expert evidence assessing whether a claimant belongs to a vulnerable minority and would be at risk if deported to their home country. This is authoritatively articulated:

‘Firstly, most Ashraf could be picked out by their appearance, being relatively light-skinned.’ (*HH & others (Mogadishu: armed conflict: risk) Somalia CG v The Secretary of State for the Home Department* [2008] UKAIT 00022, 2007 WL 7239189, para 214)

‘Individuals of non-Arab Darfuri or Nuba ethnic origin are both identifiable because of their darker skin colour and have historically been looked down upon by lighter-skinned Sudanese from tribes in central Sudan…’ (*KAM (Anonymity Direction Made) v The Secretary of State for the Home Department* [2020] UKUT 269 (IAC), 2020 WL 05548776, para A47)

Both of these cases involved claims that deporting the appellants would put them at risk of persecution because of their ethnicity. In both instances, expert evidence helped to make the decision on whether the claims were persuasive. Expert evidence plays a contested role in the adversarial court system because it involves a party paying for a written report or oral testimony from an expert of their choice. All of the parties to a case can take advantage of this (Mnookin, 2008). The expert’s evidence is an “opinion” rather than fact. However, the context behind these experts’ statements tells us several things. Looking at the first case of *HH*; here, evidence was provided by two experts. Their involvement was commissioned by the appellants to support their claims. Interestingly, in an earlier asylum case which also involved Somali appellants, one of the experts who testified in *HH* confirmed the following about himself:

‘…despite not having visited the country since 1992 he was able to keep abreast of Somali affairs through reading academic and non-academic reports, relevant websites, his discussions with FCO staff and a number of personal contacts inside and outside Somalia.’ (*NM and Others (Lone women – Ashraf) Somalia CG* [2005] UKIAT 00076, para 75)

Both experts in *HH* were white anthropologists from the UK who had undertaken ethnographic fieldwork in Somalia. They have been recognised for their contributions to asylum claims (The Editors, 2013). Similarly in *KAM* which was heard nearly 15 years after *HH*, the three experts speaking on behalf of the appellants were from the UK or America and had diplomatic and research connections to Sudan. I am not evaluating these experts’ motives. However, it is pertinent to think about their positionality and identity in these courts. Evidence in the courts and tribunals is powerful because it is part of the legitimate narrative and facts of a party’s case (Gonzales Rose, 2017). But the power of evidence and how it comes before the courts where minoritised people and issues are concerned has not been given much notice. Gonzales Rose argues that the lived realities of racialised minorities are often excluded in the courts and when they are admitted into evidence, it is “through expert witnesses’ white or “insider” voices” (Gonzales Rose, 2017, p. 2244). The authority of white experts on the claims of racialised minorities is taken for granted, even when they have not been to the place they are testifying about in over a decade. There is a power relationship being expressed here where a white expert who has studied Somalia or Sudan can make authoritative claims about the place and its people. The added weight of their white voice is needed to make the case of these vulnerable asylum-seeking appellants persuasive. They are therefore, feeding into the orientalist approach that ensures appellants lack credibility until a white person speaks on their behalf. They authorise views of Somalia and Sudan, they speak for these countries and their people who are not allowed to speak for themselves.

This flows into the greater narrative arc surrounding legal and judicial responses to asylum and immigration. The hostile environment which is designed to make these racially-minoritised people especially vulnerable requires the authoritative white and/or insider expert to support their claims. Asylum policy in the UK is guided by deterrence rather than human rights protection (Fekete, 2001). Intrinsic in this is the absorbing of asylum seekers and refugees into the broad category of migrants. Refugees and asylum seekers fleeing persecution and suffering are now viewed as immigrants (read economic migrants) (Goodman et al., 2017). Their claims are viewed with suspicion by default. When these cases came before the immigration tribunals, the Home Office had already decided that these appellants’ claims lacked merit. When you are already being treated with hostility by the government, how do you overcome their authoritative narrative and decision? By having an “insider” speak on your behalf. When you tell your story, they do not believe you. But when someone who looks and speaks like them tells your story, it makes a difference. The problem is that the authoritative insider is still exactly that: an insider who forces you to stay in the outsider position. You remain the chancer who is trying to stay without permission whilst they tell your story through their lens for a fee. Their lens which cannot be separated out from their institutionalised whiteness, or their position as defenders of white authority. Their price goes beyond monetary value; it perpetuates the norms of the hostile environment. It never allows the racialised othered asylum seeker to take control of their narrative and be taken seriously in their own voice.

Thinking more directly about the comments on skin tone, in *HH* it is claimed by the first expert that members of the Ashraf minority in Somalia can be identified by their appearance because they are relatively light-skinned. The statement is qualified by the idea that “most” rather than all Ashraf can be picked out by their lighter skin. However, this is later limited by the second expert in the case stating that:

‘skin colour is not itself determinative and…there are exceptions both ways’ (*HH* (2007), para 289)

This is later followed by the tribunal’s conclusion for one of the appellants that:

‘actual skin colour is plainly not a factor that can advance her case’ (*HH* (2007), para 360)

So, in the end, “skin colour” was not that important in making the decision on whether the appellants were from the Ashraf minority in Somalia and therefore at risk of persecution if deported. Arguably, the comments on skin tone form one small detail in the larger context of the opinions on identifying members of this vulnerable community. In any event, “skin colour” is not determinative in the tribunal’s ruling so we can forget about it there. In *KAM*, members of the Darfuri or Nuba communities are supposedly identified by their darker skin. These remarks are hidden within over 100 pages of expert evidence appended to the judgment. You would not even notice them unless you were looking for them. Does that mean they are insignificant? I argue that we should not be dismissing their presence in these official judicial records.

Their presence tells us that it is acceptable to use skin tone when determining a person’s ethnic identity and therefore their difference from members of another group. We have seen the horrifying consequences of this before. In Rwanda, European pseudo-scientific racism was used to distinguish between communities based on their physical features including their skin tone to create a hierarchy. This hierarchy positioned Tutsi and their supposed lighter skin as superior to Hutu thereby framing them as distinct groups which divided communities. Around 1 million people, mostly Tutsi were killed during the genocide in Rwanda (Baisley, 2014). Classifying people and groups based on skin tone is dangerous and reductive. The ease with which these discourses rooted in that imperialist racist science are being reproduced in the courts and tribunals today is concerning. The indifference toward this weaponisation of skin tone in asylum and immigration cases evinces a deliberate lack of care masquerading as a lack of awareness. It does not matter that these comments are irrelevant to the outcome of an appeal, what matters is that they are making it into these spaces at all. What matters is who is making these comments and their position. The white insider expert who is not a member of the affected appellant’s community is making these authorised claims of the other. There may not be a lot of weight placed on this evidence or expert opinion, but this highlights how structural and normalised the reduction of racialised people’s identities are in immigration law within the hostile environment. It is a piece of the bigger picture which is going unnoticed – an example of blackboxing which conceals the injustices around the use of legal power to oppress the minoritised. The law is blind to structural forms of oppression whether racism or colourism and there is no desire to recognise or remedy this.

Another issue concerns the social meaning attached to skin tone. Earlier I discussed how colourism is a standalone oppression because it describes how the social meaning attached to skin tone itself leads to disadvantageous treatment. In both *HH* and *KAM* we are told by the experts that skin tone as an indicator of ethnic group belonging could lead to minoritisation and persecution for the appellants. The Ashraf in Somalia are usually picked out by their relatively lighter skin; the Darfuri or Nuba in Sudan by their darker skin which leads to them being looked down upon. The evidence tells us that people experience colourism as part of their wider oppression. But we are told this in passing. The colourism is an afterthought and part of the status quo. We do not explicitly identify people through their skin tone in this jurisdiction, so it is unimportant. I am not saying that skin tone should be used as evidence in asylum applications but the normalisation of its inclusion in expert opinions is troubling. It denotes an orientalist perception of colourism as an established fact in those countries and communities. The courts are accepting that these people experience mistreatment because of their ethnic group which is marked on their bodies through their skin tone. They unquestioningly accept that it is a form of prejudice which happens in those foreign countries to divide and categorise people according to their skin tone. This lack of engagement with skin tone and by extension colourism echoes the attitudes displayed in the earlier US case of *Ali*. Colourism is not an issue for the courts in England or Britain or the UK because it does not happen here. And if it does not happen here, then there is no need to waste resources on trying to understand it. And even if it was happening in the UK, it happens in those racially minoritised communities only. In which case, it is so complex and “other,” legal actors cannot understand it, so they do not see a need to address it.

## Concluding thoughts

This was not exactly a paper about legal and judicial responses to colourism in the UK. For that to be possible, there would need to actually be some engagement with colourism in the law and courts around the UK. But colourism is not accounted for or recognised in the law here. This is mirrored in wider legal, policy and social approaches to racism. There is very little that brings attention to this form of oppression in the UK and we have only recently started to see a more robust exploration of it. As a result, I had to think more laterally and interrogate the gap between law and colourism. As I stated earlier, gaps and silences are equally informative when considering the experiences of racially-minoritised people in the UK. They tell us how and where the state and its institutions deploy their preferred legal strategy of indifference. A strategy that was honed and used to great effect by colonial administrations to dismiss, dehumanise and detach from the colonised people they were oppressing. These same attitudes are seen here as we are forced to look at how the law and courts treat skin colour more broadly.

I looked at potential spaces within existing antidiscrimination and hate offences law where victims of colourism might find a remedy. However, there was little to be found and the limitations of the statutory framework not just for colourism but more fundamentally racism were apparent from the beginning. Could something be achieved in the courts instead? Going through dozens of case reports which had no mention of colourism, I was forced to expand my analysis out to think about the ways that judicial fora consider skin colour or tone. I found repeatedly that there is an orientalist weaponisation of skin tone across different areas of law. Skin colour and tone are used and abused in the courts and legal processes to dismiss and reduce racially-minoritised people to the surface of their skin. Their skin tone or colour are used to make it easier for them to be identified as criminal offenders; to humiliate them in proceedings which are meant to give them a voice; to determine whether they deserve to be seen as a victim of persecution and can therefore seek asylum. By allowing the unchallenged use of skin tone and colour to continue like this in legal and judicial processes, racially-minoritised people are being forced to suffer even further.

In this way, indifference is a form of jurisdictional control whereby legal power does not just control what is recognised in law but *how* it is recognised: whether this involves failing to understand the specifics of colourism and the implications of identifying people through their skin tone; reducing people’s claims of suffering race discrimination to an irrelevant discussion about how light their skin is compared with a white person’s; or even by farming out the identification of a persecuted group to “experts.” This leads to my final conclusion. That the differential treatment of people based on their skin tone and colourism exist in the UK, but the problem goes beyond this. The black box of unrecognised colourism in the law has the potential to reveal much more about how colonial discourses are constructed and maintained in law for racism as well. It tells us that the operation of legal power is wielded in specific ways to dehumanise those of us who are already suffering from multiple and combined forms of marginalisation. Without opening that black box which hides colourism from the legal gaze, we can neither challenge its operation, nor can we start to explore how the law could otherwise be constructed. Thus, there is no immediate legal reform to propose: instead, I want us to seek out and open the black boxes which hide us and our pain from the law. Taking that first step will open up more possibilities for repair in the law and beyond.

## Data Availability

The original contributions presented in the study are included in the article/supplementary material, further inquiries can be directed to the corresponding author.

## Footnotes

^1^Colourism featured in the working title of the documentary, but this was dropped because of the negative reaction (see Rosseinsky, 2023).

^2^Skin tone is defined as the “colour of the surface of someone’s skin” (Cambridge Dictionary, 2025).

^3^The terms “racialised” and “racially minoritised” refer to the marginalisation, exclusion, or different treatment of people because they are not white and/or perceived to be white (El-Enany, 2020).

^4^I refer to the UK throughout this piece when making general comments about the law and wider social context. British legal responses cover England, Wales and Scotland. When I talk about law in England and Wales, this is because in those situations Scotland and Northern Ireland have their own frameworks (Hand et al., 2015).

^5^For a detailed explanation and commentary of hate crimes law, see Law Commission (2021). The Scottish legal framework is similar to that of England and Wales. See Hate Crime and Public Order (Scotland) Act 2021.

^6^Before this, in *Abbey National PLC v Chagger* (2009) I.C.R. 624, it was held that the EU’s Council Directive 2000/43/EC on race equality was intended to cover discrimination on the ground of colour because “such discrimination is in practice necessarily an aspect or manifestation of discrimination based on racial and ethnic origins” (para 35). So, colour has long been a marker of race rather than attracting distinct forms of mistreatment.

^7^Here, I am referring to the socially-constructed concept of white, rather than the physical colour. It is difficult to argue that including white as a colour was originally or solely intended to protect minoritised people with albinism.

^8^See Equality Act (2010), s13 and s19.

^9^In Scotland, the Lord Advocate’s guidelines on identification parades state that “the suspect should be placed beside persons of similar age, height, dress and general appearance” (Crown Office and Procurator Fiscal Service, 2019).

^10^My use of the term “prostitution” is deliberate to convey the orientalist portrayals of geisha, not because I am conflating the work of geisha with prostitution (or sex work for that matter). Nor am I commenting on prostitution and sex work but the mainstream negative connotations around them.
